# Comparative analysis between endometrial proteomes of pregnant and non-pregnant ewes during the peri-implantation period

**DOI:** 10.1186/s40104-015-0017-0

**Published:** 2015-04-25

**Authors:** Haichao Zhao, Linlin Sui, Kai Miao, Lei An, Dong Wang, Zhuocheng Hou, Rui Wang, Min Guo, Zhilong Wang, Jiqiang Xu, Zhonghong Wu, Jianhui Tian

**Affiliations:** Ministry of Agriculture Key Laboratory of Animal Genetics, Breeding and Reproduction, National engineering laboratory for animal breeding, College of Animal Sciences and Technology, China Agricultural University, No.2 Yuanmingyuan Xi Lu, Haidian, Beijing, 100193 China; The Key Laboratory for Farm Animal Genetic Resources and Utilization of Ministry of Agriculture of China, Institute of Animal Science, Chinese Academy of Agriculture Sciences, Beijing, 100193 China; National Engineering Laboratory for Animal Breeding and MOA Key Laboratory of Animal Genetics and Breeding, China Agricultural University, Beijing, 100193 China; College of Animal Science and Technology, Agricultural University of Hebei, Baoding, Hebei 071000 People’s Republic of China; State Key Laboratory of Animal Nutrition, College of Animal Sciences and Technology, China Agricultural University, No.2 Yuanmingyuan Xi Lu, Haidian, Beijing, 100193 China; Department of Biochemistry and Molecular, Dalian Medical University, Dalian, 116044 China

**Keywords:** Early pregnancy loss, Endometrium, Ewe, LC-MS/MS, Proteome

## Abstract

**Background:**

Early pregnancy failure has a profound impact on both human reproductive health and animal production. 2/3 pregnancy failures occur during the peri-implantation period; however, the underlying mechanism(s) remains unclear. Well-organized modification of the endometrium to a receptive state is critical to establish pregnancy. Aberrant endometrial modification during implantation is thought to be largely responsible for early pregnancy loss.

**Result:**

In this study, using well-managed recipient ewes that received embryo transfer as model, we compared the endometrial proteome between pregnant and non-pregnant ewes during implantation period. After embryo transfer, recipients were assigned as pregnant or non-pregnant ewes according to the presence or absence of an elongated conceptus at Day 17 of pregnancy. By comparing the endometrial proteomic profiles between pregnant and non-pregnant ewes, we identified 94 and 257 differentially expressed proteins (DEPs) in the endometrial caruncular and intercaruncular areas, respectively. Functional analysis showed that the DEPs were mainly associated with immune response, nutrient transport and utilization, as well as proteasome-mediated proteolysis.

**Conclusion:**

These analysis imply that dysfunction of these biological processes or pathways of DEP in the endometrium is highly associated with early pregnancy loss. In addition, many proteins that are essential for the establishment of pregnancy showed dysregulation in the endometrium of non-pregnant ewes. These proteins, as potential candidates, may contribute to early pregnancy loss.

**Electronic supplementary material:**

The online version of this article (doi:10.1186/s40104-015-0017-0) contains supplementary material, which is available to authorized users.

## Introduction

Pregnancy loss in livestock and humans is estimated to occur in 20 to 40% of pregnancies [1], two-thirds of which occur during the peri-implantation period [2,3]. Implantation, a critical event for establishing pregnancy, involves an elaborate set of molecules essential to maternal–fetal crosstalk. Ruminants have been used extensively to investigate maternal–fetal interactions during implantation [4]. As a biological sensor of the embryo, well-organized modification of the uterine endometrium is critical for successful implantation [5]. Aberrant endometrial modifications during implantation are believed to be largely responsible for failed pregnancy establishment [6].

Despite many previous studies on factors involved in early pregnancy loss, the underlying mechanism(s) remains unclear. The ‘-omic’ approaches, including RNA-seq, microarray and proteomic profiling, have been used to profile the gene or protein expression patterns in the endometrium during peri-implantation period. Mansouri-Attia showed gene expression patterns in the bovine endometrium during early pregnancy after artificial insemination (AI) using microarray analysis [7]. Similarly, transcriptomes of the endometrium during early pregnancy have also been profiled in ewes [8], humans [9], mice [10], pigs [11] and horses [12]. However, these studies, in which the non-pregnant controls were cyclic female animals [7,8,13], animals inseminated with the supernatant of centrifuged sperm [14], or recipients that received a sham transfer [15], have generally focused on characterizing expression profiles for the establishment of pregnancy, and the results are not suitable to characterize the endometrial proteomic profile in ruminants undergoing early pregnancy loss.

Using ewes as model, the aim of our study was to establish a proteomic database to study the characteristics of the endometrium associated with early pregnancy loss. The endometrial proteomic profiles were compared between pregnant and non-pregnant (failed pregnant) ewes on Day 17 of pregnancy. , It should be mentioned that this time point was documented as the end point of the peri-implantation period in sheep [16,17], and also extensively selected in previous studies to explore endometrium involved mechanisms of pregnancy establishment [18-21].

Compared with transcriptomic analysis by RNA-seq or microarray, the proteomic approach has the major advantage of investigating functional molecules directly. To the best of our knowledge, this study provides the first endometrial proteomic profile for ewes, as well as the first liquid chromatography/electrospray ionization tandem mass spectrometry (LC-ESI-MS/MS)-derived proteomic profile of the endometrium from a ruminant species. More importantly, our analysis implicated a number of endometrial proteins as being associated with early pregnancy loss in ewes. Our results also provided a comprehensive and detailed reference for further study of the mechanisms of early pregnancy loss.

## Materials and methods

### Experimental design

**Figure 1:**
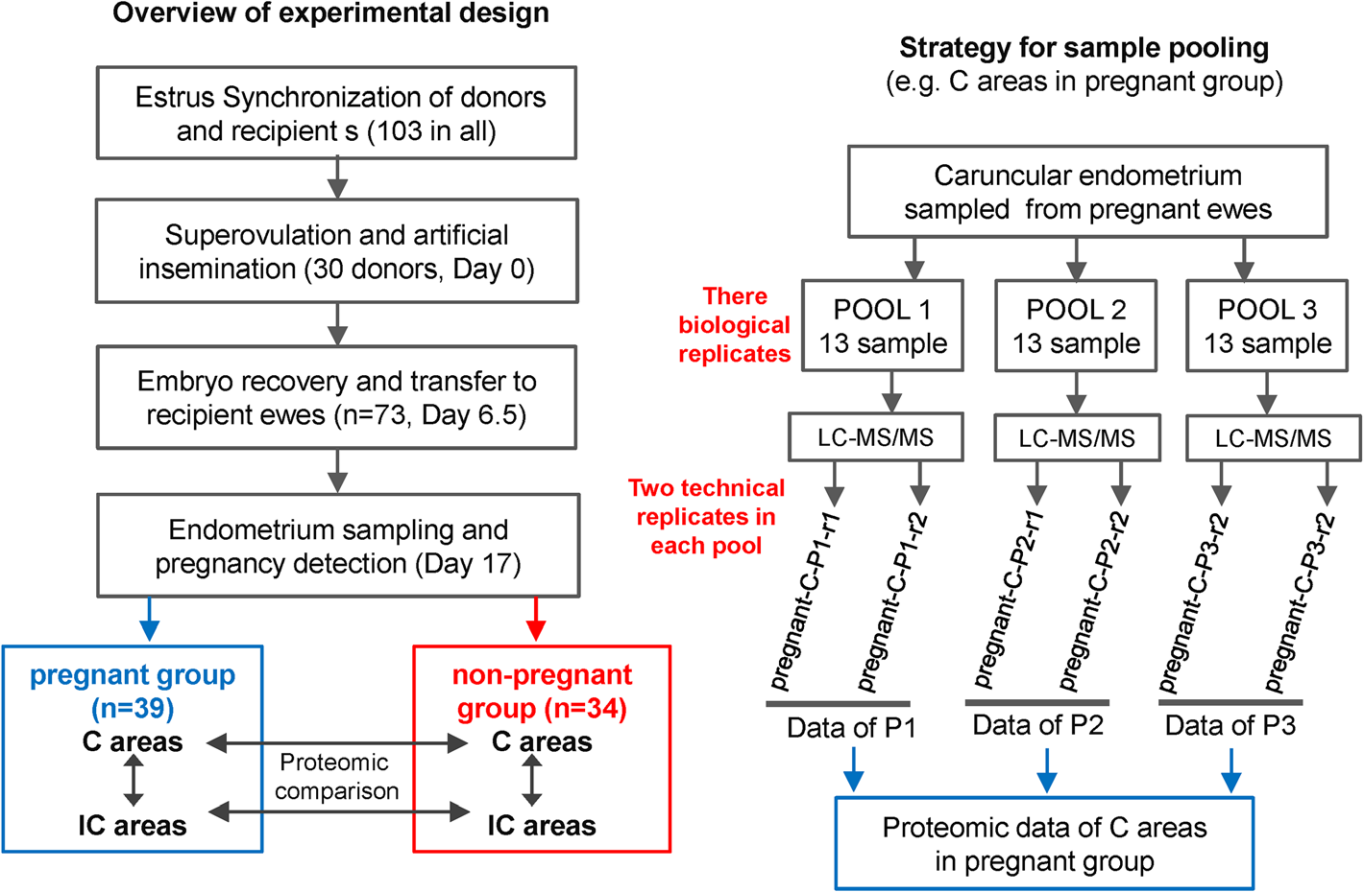
Overview of the experimental design.

As mentioned above, the aim of this study was to establish an endometrial proteome that characterized early pregnancy loss, and to provide a reference for studying the endometrium’s involvement in pregnancy loss. For this purpose, ewes that received embryo transfer were assigned as pregnant and non-pregnant groups, based on the presence or absence of well-developed filamentous conceptus on Day 17 of pregnancy (Figure 1). Considering the fact that the outcome of pregnancy could be influenced by various factors, all experimental procedures, including the management of donor and recipient ewes, *in vivo* embryo production and transfer, as well as conceptus detection and sample collection, etc., were performed under strictly controlled and unified conditions, therefore, the endometrial proteome of non-pregnant ewes would largely reflect the characteristics that are associated with, or responsible for, early pregnancy loss. In addition, considering that both pregnant and non-pregnant ewes had received well-developed embryos, and all experimental procedures were performed under strictly controlled and unified conditions, we considered that the outcome of pregnancy was largely caused by the different response and modulation of the maternal uterus to the transferred embryos.

The C areas of the endometrium are the sites of superficial implantation and placentation in ewes, while the IC areas contain large numbers of branched and coiled uterine glands that synthesize, secrete and transport a variety of molecules essential to the development of conceptus [22]. Given the differences in structure and biological functions associated with the C and IC areas, these two distinct endometrial zones are always analyzed separately in detailed studies of the implantation process [5,23]. Therefore, a global proteomic analysis of the endometrium was carried out to characterize the endometrial protein expression patterns associated with early pregnancy loss. We first established proteomic profiles of the C and IC areas between pregnant and non-pregnant ewes; thereafter, we compared the profiles between the C and IC areas of pregnant and non-pregnant ewes (Figure 1), as in a previous study [7]. Endometrial samples from each ewe in the same group were divided into three pools as biological replicates, and each pooled sample was divided into two equal aliquots and processed as technical replicates. Data for each pool were obtained by taking the average of results from the two aliquots (Figure 1).

### Animals and treatments

Procedures for handling animals were in accordance with the Guide for the Care and Use of Agricultural Animals in Agricultural Research and Teaching, and the Animal Use Committee, China Agricultural University, approved all the procedures. Chinese Small Tail Han ewes with normal ovarian cycles (n = 103) were selected after general clinical examinations. All animals were fed and managed under unified and optimized conditions of environment and nutrition, before and after embryo transfer. The estrous cycle was synchronized using progesterone-impregnated (0.3 g) vaginal implants with controlled intra-vaginal drug release (CIDR-B^TM^, Pfizer Animal Health, Auckland, New Zealand) for 13 d. The recipients (n = 73) each received an intramuscular (i.m.) injection of 15 mg of prostaglandin F_2α_ (Lutalyse, Pfizer, New York, NY, USA) 2 d before the progesterone vaginal implant was removed. The donors (n = 30) were administered with follicle stimulating hormone (Folltropin-V; Vetrepharm Canada Inc.; Belleville, ON, Canada) i.m. at doses of 40 mg, 30 mg, 30 mg and 24 mg at 12-h intervals to achieve multiple ovulations, beginning 48 h before progesterone withdrawal. Three artificial inseminations were performed within a 12-h interval, beginning 24 h after the progesterone vaginal implant was removed. The day of progesterone withdrawal was defined as Day 0. Introducing and recovering 500 mL phosphate buffered saline (PBS) into each uterine horn at 6.5 d post-insemination flushed the blastocysts. A stereomicroscope was used to perform embryo biopsies. Following the biopsy, two well-developed blastocysts were transferred per synchronized recipient ewe to ensure normal pregnancy signals during implantation. To minimize the difference between pregnant and non-pregnant ewes contributed by the surgical procedure and embryo quality, we selected a single skillful technician, and only good-quality (referred as Grade 1) blastocysts were transferred. By this method, it is accepted that the outcome of pregnancy could be mostly attributed to the response and modulation of the maternal uterus to the transferred embryos.

### Endometrial tissue recovery

The recipients were sacrificed on Day 17 of pregnancy, and their uteri were collected and flushed with PBS. Animals with filamentous conceptus were assigned to the pregnant group, while ewes without a visual conceptus were included in the non-pregnant group. The presence of a conceptus was obvious because there was an apparently normal elongated conceptus of 25 cm in length or more attached to the endometrium of the pregnant ewes by Day 17 [24], while no such filamentous conceptus was observed in the non-pregnant group. Samples of the endometrial caruncular (C) and intercaruncular (IC) areas were taken and processed as described by Mansouri-Attia [5]. Scissors were used to open the ipsilateral uterine horn longitudinally. Endometrial C area tissues were first carefully cut out, and then the endometrial IC areas were sampled. The tissues were sampled from all the animals by the same technician and stored at −80°C until further analysis.

### Protein extraction

A sample from each aliquot prepared as described above was ground to powder in liquid nitrogen and stored overnight at −20°C after adding a five-fold volume of chilled acetone containing 10% trichloroacetic acid (TCA) and 10 mmol/L dithiothreitol (DTT). The samples were then centrifuged at 4°C, 16,000 × *g* for 20 min and the supernatant discarded. The precipitates were mixed with 1 mL chilled acetone containing 10 mmol/L DTT and centrifuged at 4°C, 20,000 × *g* for 30 min after storing for 30 min at −20°C. Centrifugation was repeated several times until the supernatant was colorless. The pellets were air-dried, dissolved in lysis buffer containing 1 mmol/L phenylmethanesulfonyl fluoride (PMSF), 2 mmol/L ethylenediaminetetraacetic acid (EDTA) and 10 mmol/L DTT and sonicated at 200 Watts for 15 min before being centrifuged at 30,000 × *g* at room temperature for 30 min. The Bradford method was used to determine the protein concentration of each supernatant.

### Peptide digestion

Fifty micrograms of protein was taken from each sample, and isopycnic samples were prepared by adding 8 mol/L urea solution. To reduce disulfide bonds, the samples were incubated with 10 mmol/L DTT at 56°C for 1 h, and then cysteine bonding was blocked using 55 mmol/L iodoacetamide (IAM) in a dark room for 45 min. Thereafter, each sample was diluted 8-fold with 50 mmol/L ammonium bicarbonate and digested with Trypsin Gold at a protein: trypsin ratio of 20:1 at 37°C for 16 h. Following desalting using a Strata × C18 column (Phenomenex), the samples were vacuum dried.

### LC-ESI-MS/MS analysis with LTQ-orbitrap collision induced dissociation (CID)

Each sample was resuspended in buffer A [2% acetonitrile (ACN), 0.1% formic acid (FA)] and centrifuged at 20,000 × *g* for 10 min. The final peptide concentration for each sample was approximately 0.5 μg/mL. The digested samples were fractionated using a Shimadzu LC-20AD nano-HPLC system. Each sample (10 μL) was loaded by the autosampler onto a 2-cm C18 trap column (200-μm inner diameter), and the peptides were eluted onto a resolving 10-cm analytical C18 column (75-μm inner diameter) prepared in-house. The samples were loaded at a flow rate of 15 μL/min for 4 min, and then a 91-min gradient from 2% to 35% buffer B (98% ACN, 0.1% FA) was run at a flow rate of 400 nL/min, followed by a 5-min linear gradient to 80% buffer B that was maintained for 8 min before finally returning to 2% buffer B within 2 min. The peptides were subjected to nanoelectrospray ionization and then detected by MS/MS in an LTQ Orbitrap Velos (Thermo Fisher Scientific, Bremen, Germany) coupled online to a high performance liquid chromatography (HPLC) system. Intact peptides were detected in the Orbitrap analyzer at a resolution of 60,000. Peptides were selected for MS/MS using the CID operating mode with a normalized collision energy setting of 35%, and ion fragments were detected in the LTQ. One MS scan followed by ten MS/MS scans was applied for the ten most abundant precursor ions above a threshold ion count of 5,000 in the MS survey scan. Dynamic exclusion was used, and the parameters were set as follows: repeat counts = 2; repeat duration = 30 sec; and exclusion duration = 120 sec. The applied electrospray voltage was 1.5 kV. Automatic gain control (AGC) was used to prevent overfilling of the ion trap; 1 × 10^4^ ions were accumulated in the ion trap for generation of CID spectra. For MS scans, the m/z scan range was 350 to 2,000 Da.

### Proteomic analysis

MaxQuant software (version 1.1.1.36) was used to analyze the mass spectra, which generates a list of peptide peaks for searching a reference protein database and evaluates statistically the results of protein identification and quantitation based on computational algorithms from Cox and Mann [25]. The cow is the only well-annotated species with a genomic database with a high degree of homology to sheep. Therefore, we generated one reference protein database by integrating the following databases and sequences of cow proteins and limited publically available sheep proteins and removed duplicate proteins: GenBank nr (20110403), Uniprot cow proteins (20110503), sheep proteins (http://www.livestockgenomics.csiro.au/sheep/) and cow proteins (http://genomes.arc.georgetown.edu/drupal/bovine/). The search engine embedded in MaxQuant was used to search the MS/MS data against the reference protein database. Up to two missed cleavages were allowed. The first search was set to 20 ppm, and the MS/MS tolerance for CID was set to 0.5 Da. The false discovery rate (FDR) was set to 0.01 for peptide and protein identifications. Proteins were considered identified when at least two peptides were identified, at least one of which was uniquely assignable to the corresponding sequence. In the case of identified peptides that were all shared between two proteins, these were combined and reported as one protein group. Contents of the protein table were filtered to eliminate identifications from the reverse database and common contaminants. The minimum peptide length was set to six amino acids. A minimum of two peptides, with one being unique, was required for protein identification. The MaxQuant software suite containing an algorithm based on the extracted ion currents (XICs) of the peptides was used to perform label-free quantification analysis. This specific label-free processing method described by Waanders *et al.* [26] has been successfully applied in other label-free quantification studies [27].

### Data analysis

To facilitate data analysis, all proteins were mapped to the Ensembl *Bos taurus* gene ID. Student’s t-test was used to detect the significance of the differentially expressed proteins (DEPs), and *P* < 0.05 was considered significant. DAVID version 6.7 (http://david.abcc.ncifcrf.gov/) enables the generation of specific functional annotations of biological processes affected by treatment from the target gene lists produced in high-throughput experiments [28,29]. We used the DAVID platform to annotate biological themes for the different groups (pregnant vs. non-pregnant ewes) or different endometrial locations (C vs. IC areas). Moreover, gene symbols corresponding to DEPs were also sent to the Search Tool for the Retrieval of Interacting Genes/Proteins (STRING version 9; http://string.embl.de/) to build a network using edge information from three separate forms of evidence: databases, experiments and text mining. We used 0.4 (medium confidence) as the value for edge confidence provided by STRING. To assess the similarities of the different replicates, and to obtain a visual understanding of the relationship between the different experimental groups, the CLUSTER 3.0 data analysis tool carried out hierarchical clustering based on the clusters of protein expression profile of different technical and biological replicates.

## Results

### Summary of the endometrial proteome from pregnant and non-pregnant ewes

Overall, 73 recipient ewes underwent the embryo transfer procedure. Among them, 39 ewes supported blastocyst development to an elongating conceptus, and 34 experienced failed early pregnancy. We successfully identified 7494, 8216, 7459, 7933 peptides, and 1486, 1569, 1494 and 1574 proteins in the C and IC areas of non-pregnant ewes, as well as C and IC areas of pregnant ewes, respectively (Additional file 1: Table S2-S5, S10).

The precision of quantitation between the technical replicates was evaluated by Pearson’s correlation coefficient, as in a previous study [30]. As shown in Additional file 2: Table S1 and Additional file 3: Figure S1, we found an average PCC (Pearson correlation coefficients) of 0.9886 for the protein level and 0.9492 for the peptide level. According to the criteria proposed in Waanders et al.’s study (0.87 and 0.98 for peptide and protein respectively) [26], the correlation values between technical replicates should be sufficient.

Hierarchical clustering was performed based on the overall similarity of protein expression patterns of different groups and replicates. The results showed a striking separation of pregnant and non-pregnant samples into major opposing branches for both the C and IC areas (Additional file 4: Figure S2). In both areas, the endometrial proteomes of the non-pregnant ewes were very distinct from those of pregnant ewes. In addition, technical replicates in each pool were tightly clustered in the same branch, confirming the reliability of our detection system.

Additional file 5: Figure S3 shows the distribution of DEPs (pregnant *vs*. non-pregnant) with different fold changes in the C and IC areas. It should be noted that in both the C and IC areas, more DEPs showed significant downregulation in non-pregnant ewes.

### Analysis of proteomic profiles of pregnant vs. non-pregnant ewes in endometrial C area

**Figure 2:**
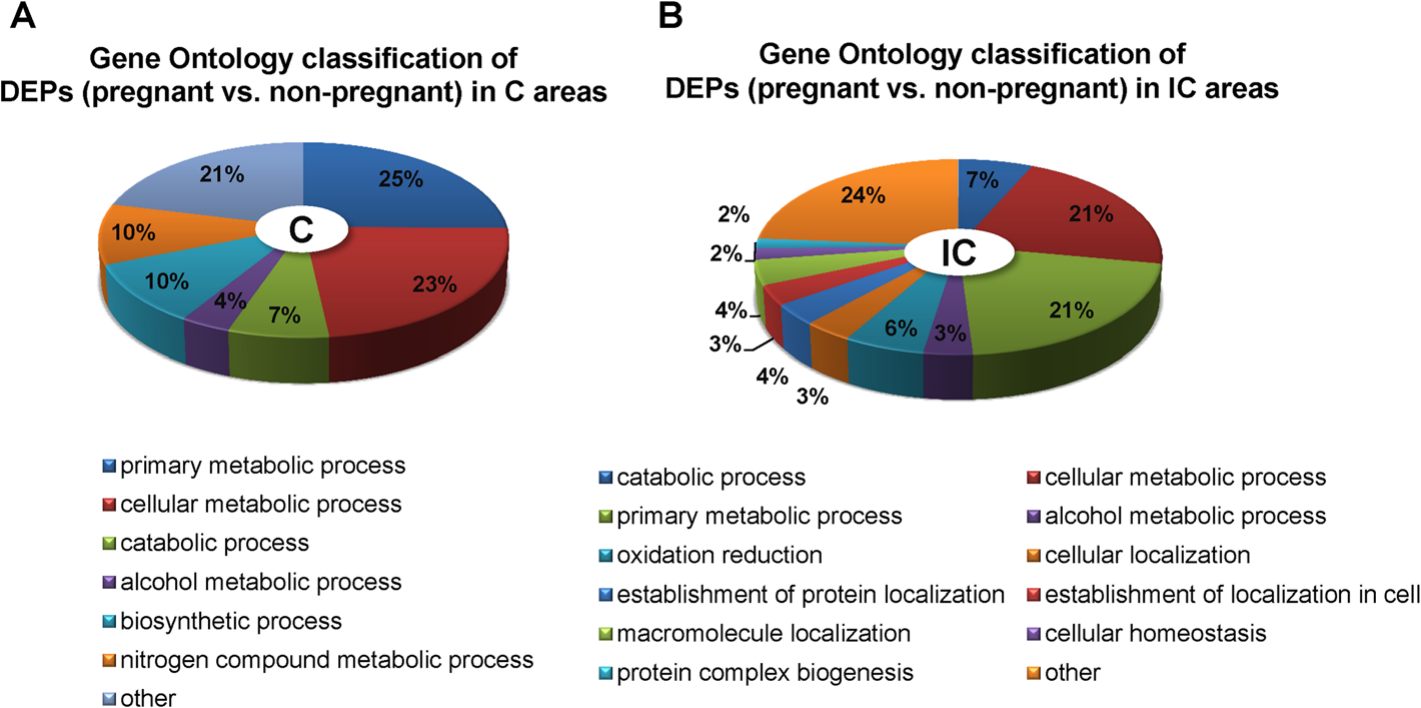
Gene Ontology “biological process” classifications of differentially expressed proteins (DEPs) (pregnant *vs*. non-pregnant) in the endometrial **A)** caruncular (C) and **B)** intercaruncular (IC) areas.

By comparing the abundance of proteins in the C areas, 94 DEPs (*P* < 0.05) were found between pregnant and non-pregnant ewes (Additional file 1: Table S6). Among these DEPs, 14 proteins were more abundant in non-pregnant ewes, and 80 were upregulated in pregnant ewes. In the C area, the dramatically increased proteins (>8-fold) in the pregnant ewes included MX2 (myxovirus resistance 2, 100-fold), IFIT3 (interferon-induced protein with tetratricopeptide repeats 3, 93-fold), ISG15 (ubiquitin-like protein ISG15, 75-fold), CASP3 (caspase-3 precursor, 12.7-fold), SLC2A1 (solute carrier family 2, facilitated glucose transporter member 1, 11.9-fold), OAS2 (2′-5′-oligoadenylate synthetase 2, 9.6-fold), NCBP1 (nuclear cap-binding protein subunit 1, 9.3-fold) and MMS19 (MMS19 nucleotide excision repair homolog, 8.4-fold). In addition, we detected six proteins that were specifically expressed in the endometrial C area of pregnant ewes: SMPDL3B (acid sphingomyelinase-like phosphodiesterase 3b), UNC45A (protein unc-45 homolog A), UGDH (UDP-glucose 6-dehydrogenase), IFIT1 (interferon-induced protein with tetratricopeptide repeats 3), GALE (UDP-galactose-4-epimerase) and LOC100140733 (uncharacterized protein). To gain an insight into biological processes characterizing early pregnancy loss, the 94 DEPs in the endometrial C areas were analyzed using DAVID Bioinformatics Resources 6.7. Figure 2A shows a simplified overview of the GO (Gene Ontology) terms based on the major category of “biological process”, and seven terms were identified for subcategorization. “Primary metabolic process” and “cellular metabolic process” were the two most represented processes.

### Analysis of proteomic profiles of pregnant vs. Non-pregnant ewes in endometrial IC area

In the IC area, we detected 257 DEPs (*P* < 0.05) between pregnant and non-pregnant ewes (Additional file 1: Table S7). Among these proteins, 49 and 209 proteins were upregulated in non-pregnant and pregnant ewes, respectively. The dramatically increased proteins in the endometrial IC areas of pregnant ewes included MX2 (140-fold), IFIT3 (88-fold), ISG15 (53-fold), SPTBN4 (spectrin, beta, non-erythrocytic 4, 29.8-fold), IFIT2 (interferon-induced protein with tetratricopeptide repeats 2, 23.7-fold), TAPBP (TAP binding protein, 10.8-fold), ERAP1 (endoplasmic reticulum aminopeptidase 1, 9.2-fold), STAT1 (signal transducer and activator of transcription 1-alpha/beta, 9.1-fold), CKAP5 (cytoseton associated protein 5, 9.0-fold), PSMA4 (proteasome subunit alpha type-4, 8.2-fold), MAT2B (methionine adenosyltransferase 2 subunit beta, 8.2-fold) and DDX58 (DEAD (Asp-Glu-Ala-Asp) box polypeptide 58, 8.0-fold). In addition, we detected six proteins that were specifically expressed in the endometrial IC area of pregnant ewes: PLAA (phospholipase A-2-activating protein), GBA (glucosylceramidase precursor), STAG2 (stromal antigen 2), COG3 (component of oligomeric golgi complex 3), HSDL2 (hydroxysteroid dehydrogenase-like protein 2) and CMPK2 (cytidine monophosphate (UMP-CMP) kinase 2). The DEPs in the IC area between the two groups were also analyzed by GO categories (Figure 2B). Based on the major category of “biological process”, 12 subcategories were enriched, with “primary metabolic process” and “cellular metabolic process” being the two most enriched terms.

### Venn diagrams comparing proteomic profiles of pregnant and Non-pregnant groups

**Figure 3:**
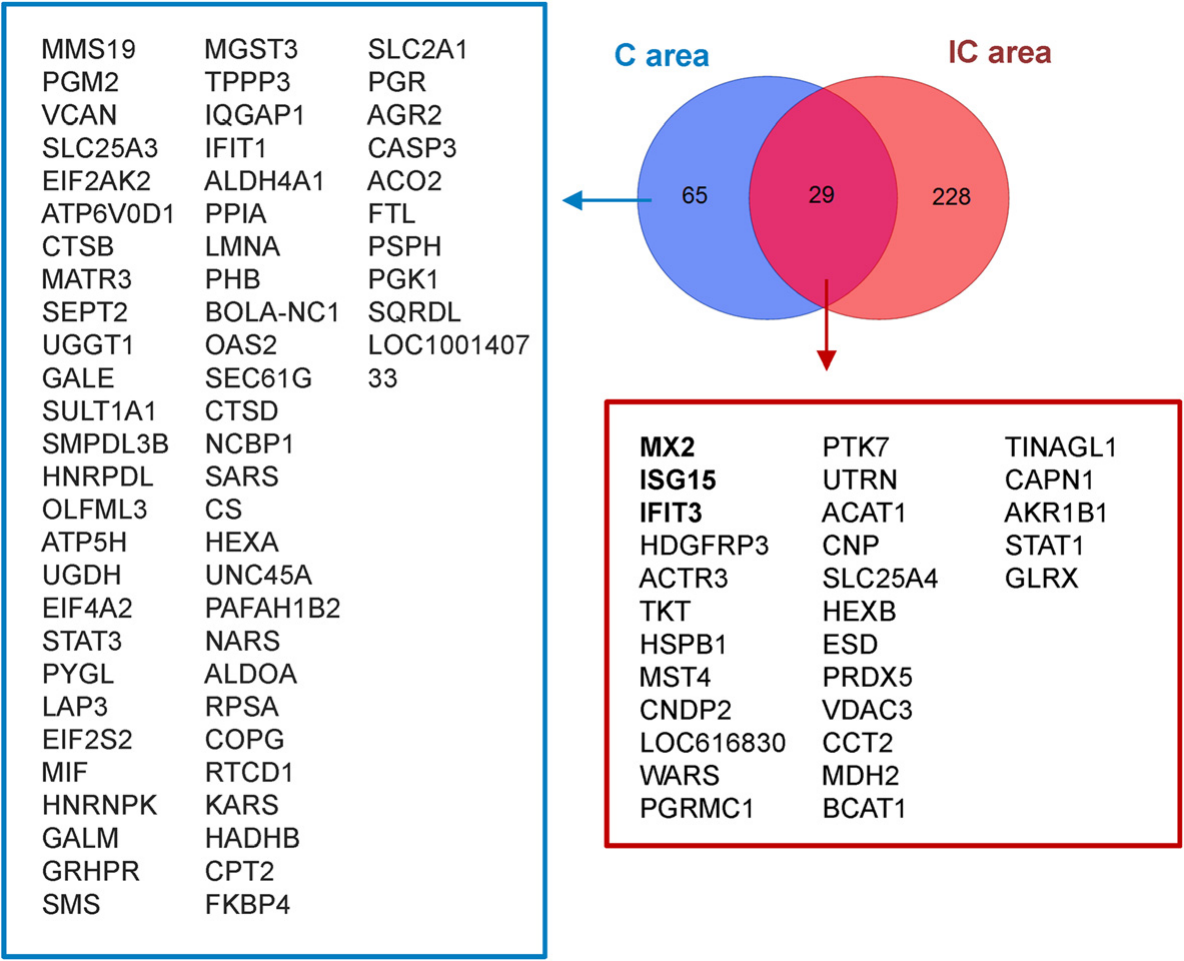
Venn diagram of DEPs between pregnant and non-pregnant ewes either common to both or specific to C or IC areas. The left box includes 65 DEPs (pregnant *vs*. non-pregnant) specific to the C area. The lower box shows 29 DEPs between pregnant and non-pregnant groups differentially expressed in both C and IC areas. Gene symbols represent the proteins.

In the comparison of pregnant *vs*. non-pregnant groups, the Venn diagram in Figure 3 shows 65 and 228 DEPs that specifically changed in the endometrial C and the IC areas, respectively. Interestingly, MX2, IFIT3 and ISG15 were the three top altered proteins among the 29 DEPs common to the C and IC areas (Figure 3). Analysis using the DAVID bioinformatic tool indicated that many KEGG (Kyoto Encyclopedia of Genes and Genomes) pathways involved in nutrition and energy were involved in early pregnancy loss in both the C and IC areas (Table 1). These KEGG pathways included “glycolysis/gluconeogenesis”, “pentose phosphate pathway” and “citrate cycle (TCA cycle)”.

**Table 1:** **Significantly enriched KEGG pathways associated with differentially expressed proteins (DEPs) (pregnant** ***vs*** **. non-pregnant) in the endometrial caruncular (C) and intercaruncular (IC) areas**

**Comparison**	**Term**	**Count**	***P-*** **value**
pregnant vs. non-pregnant (C areas)	Amino sugar and nucleotide sugar metabolism	5	7.30E-04
	Glyoxylate and dicarboxylate metabolism	3	5.00E-03
	Aminoacyl-tRNA biosynthesis	4	7.00E-03
	Glycolysis / Gluconeogenesis	4	1.70E-02
	Pentose phosphate pathway	3	2.30E-02
	Galactose metabolism	3	2.30E-02
	Lysosome	5	2.50E-02
	Citrate cycle (TCA cycle)	3	3.70E-02
	Starch and sucrose metabolism	3	4.70E-02
pregnant vs. non-pregnant (IC areas)	Valine, leucine and isoleucine degradation	8	8.50E-05
	Pyruvate metabolism	7	2.50E-04
	Proteasome	7	6.60E-04
	Citrate cycle (TCA cycle)	6	8.60E-04
	Ribosome	9	1.00E-03
	Tryptophan metabolism	6	2.80E-03
	Propanoate metabolism	5	6.70E-03
	Glycolysis / Gluconeogenesis	6	1.10E-02
	Fatty acid metabolism	5	1.40E-02
	Oocyte meiosis	8	1.80E-02
	Glutathione metabolism	5	2.40E-02
	Glyoxylate and dicarboxylate metabolism	3	2.90E-02
	Butanoate metabolism	4	4.30E-02
	Cysteine and methionine metabolism	4	4.30E-02

Count: number of proteins associated with the term. *P*-value: Modified Fisher Exact *P*-value, EASE Score.

### Interactive network analysis of DEPs between pregnant and Non-pregnant ewes

**Figure 4:**
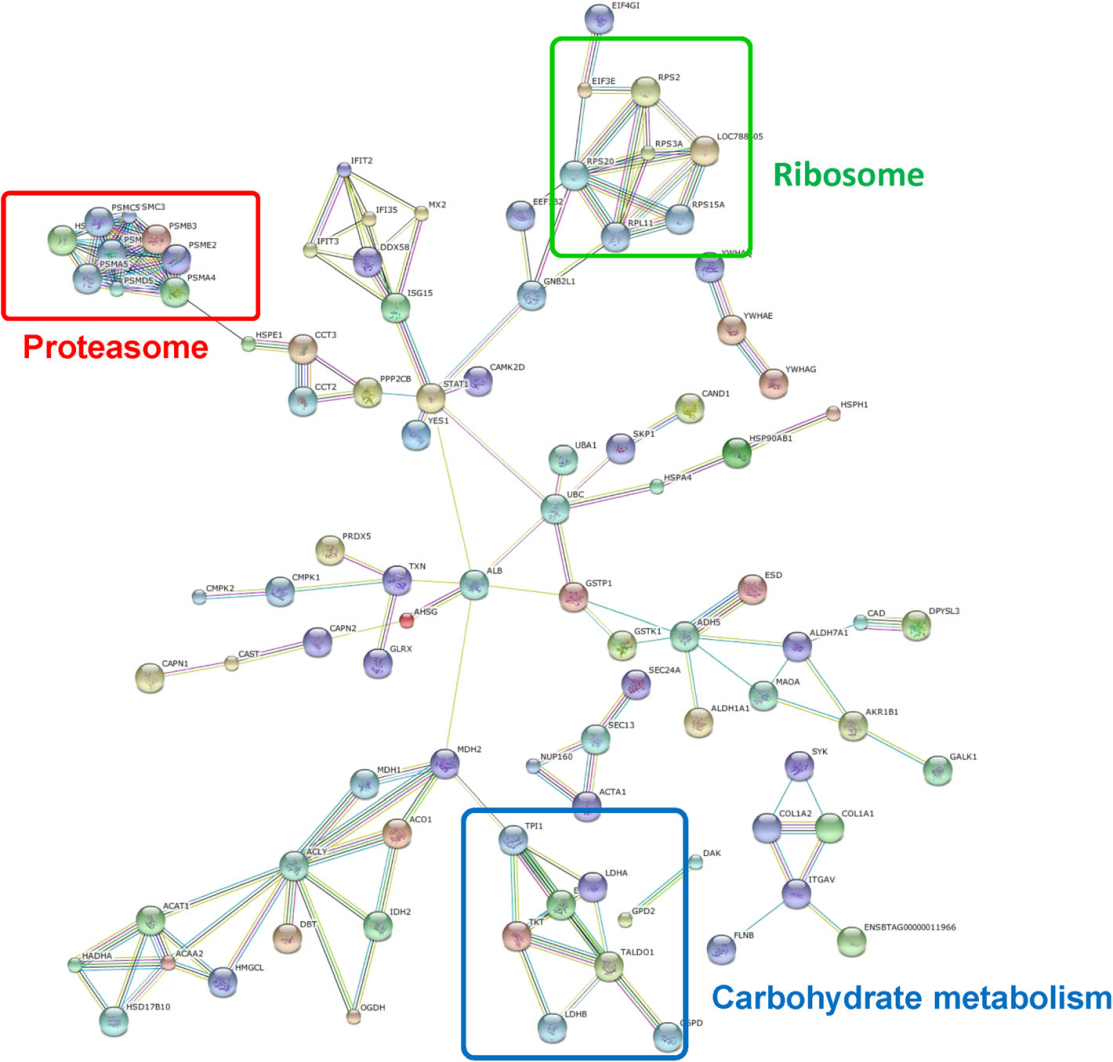
Interactive network analysis of DEPs between pregnant and non-pregnant ewes in the IC area. Proteins enclosed in color-coded outlines are mainly involved in activities of carbohydrate metabolism (blue), proteasome (red) and ribosome (green).

In the endometrial C area, 94 DEPs between pregnant and non-pregnant ewes were analyzed using the Retrieval of Interacting Genes/Proteins STRING (version 9), and a network was built using edge information. The results showed that biological processes involved in energy utilization and IFN (interferon) signaling may be associated with early pregnancy loss (Additional file 6: Figure S4). Furthermore, some DEPs (such as ISG15, MX2, OAS2 and STAT1) enriched in the network were related to IFN signaling. Additionally, many proteins, such as CS (citrate synthase, mitochondrial), ACAT1 (acetyl-Coenzyme A acetyltransferase 1), ACO2 (aconitate hydratase, mitochondrial) and MDH2 (malate dehydrogenase, mitochondrial), present in the biological process category, were related to energy utilization. In the endometrial IC area, analysis of 257 DEPs between pregnant and non-pregnant ewes indicated that these proteins were involved in major biological processes related to energy utilization [e.g., TALDO1 (transaldolase 1), G6PD (glucose-6-phosphate dehydrogenase), TPI1 (triosephosphate isomerase), MDH2], the ribosome [e.g., RPS2 (40S ribosomal protein S2), RPS20 (40S ribosomal protein S20), RPL11 (60S ribosomal protein L11), RPS15A (40S ribosomal protein S15a)] and the proteasome [e.g., PSME2 (proteasome activator complex subunit 2), PSMA4 (proteasome subunit alpha type-4), PSMA5 (proteasome subunit alpha type-4)] (Figure 4).

### Proteomic profiling of endometrial C vs. IC areas from pregnant and Non-pregnant ewes

Comparative analyses of proteomic profiles between endometrial C and IC areas within each of the pregnant and non-pregnant groups were also performed. For non-pregnant ewes, 216 DEPs (*P* < 0.05) were identified between C and IC areas (Additional file 1: Table S8). Furthermore, 191 proteins were differentially expressed (*P* < 0.05) between C and IC areas in pregnant ewes (Additional file 1: Table S9). These DEPs were also analyzed for enriched KEGG pathways and GO terms, and several of them associated with extracellular matrix (ECM)-receptor interactions and cell adhesion were enriched in the pregnant ewes, but not in the non-pregnant ewes (Table 2 and Additional file 7: Figure S5).

**Table 2:** **Significantly enriched KEGG pathways associated with differentially expressed proteins (DEPs) (caruncular (C)** ***vs*** **. intercaruncular (IC)) from pregnant and non-pregnant ewes**

**Comparison**	**Term**	**Count**	***P-*** **value**
C vs. IC areas (pregnant group)	Focal adhesion	14	2.60E-05
	ECM-receptor interaction	8	4.40E-04
	Valine, leucine and isoleucine degradation	5	8.70E-03
	Pyruvate metabolism	4	3.10E-02
	Adherens junction	5	3.50E-02
	Amino sugar and nucleotide sugar metabolism	4	4.30E-02
C vs. IC areas (non-pregnant group)	Ribosome	8	1.40E-03
	Antigen processing and presentation	7	2.20E-03
	Spliceosome	8	1.10E-02
	Proteasome	5	1.20E-02
	Pentose and glucuronate interconversions	3	2.70E-02
	Starch and sucrose metabolism	4	3.30E-02
	Phenylalanine metabolism	3	4.50E-02
	Aminoacyl-tRNA biosynthesis	4	4.60E-02

Count: number of proteins associated with the term. *P*-value: Modified Fisher Exact *P*-value, EASE Score.

## Discussion

Although aberrant molecular processes in the endometrium during implantation make a large contribution to early pregnancy loss, associated studies are limited, mainly because of the lack of a proper research model or protocol. Knockout or mutant mouse models with a pregnancy loss phenotype are generally caused by one or a few defined factors. Thus, comprehensively identifying uterine mechanisms that lead to an early pregnancy loss is challenging. It should be mentioned that, in the present study, considering that the outcome of embryo transfer would be influenced by multiple factors, transfer procedure and embryo quality were strictly controlled according a well-established procedure to minimize the contribution of other factors to the difference between pregnant and non-pregnant endometria. In addition, two well-developed blastocysts were transferred to ensure that there would be sufficient pregnancy signals derived from the conceptus during the maternal-fetal dialogue. By this methodology, the characterized protein expression patterns of the non-pregnant endometrium were largely attributed to aberrant modifications and functions of the endometrium. These systematic results provide a window into the mechanistic framework of early pregnancy loss.

### Comparison to related studies in ruminants

Our study aimed to provide a proteomic reference for understanding the mechanisms associated with early pregnancy loss. Certain previous studies were performed to profile the transcriptome of endometrium during establishment of pregnancy in ruminants [7,8] (Additional file 8: Figure S6). Here, we compared the results of our proteomic data with the finding of these studies. Some of the DEPs found in our study, such as STAT1, MX2, IFIT1and ISG15 in the C area, as well as STAT1, TUBB and COL1A2 in the IC area, were also identified as involved in the establishment of pregnancy in ruminants by previous studies [7,8]. Notably, some of these overlapping DEPs, including MX2 (100-fold in the C area and 140-fold in the IC area), ISG15 (75-fold in the C areal and 53-fold in the IC area), showed dramatic fold changes in our study, implying that these proteins may make a significant contribution to early pregnancy loss.

In the present study, the DEPs between the pregnant and non-pregnant ewes were primarily involved in immune responses, nutrient transport and proteasome-mediated proteolysis and cell adhesion, which were also significantly clustered in transcriptomic studies focusing on the establishment of pregnancy in ruminants. This indicated that aberrant expression of critical proteins and dysfunction of biological processes that are essential to establish pregnancy perhaps participate in or contribute to early pregnancy loss. However, it also should be noted the pregnant *vs*. non-pregnant DEPs that were common to previous related studies accounted for a very small part of our proteomic data. For example, five (5.32%) DEPs in the C area and five (1.95%) DEPs in the IC area are common to DEGs between pregnant (after AI) and cyclic bovines [7]; two (2.13%) DEPs in the C area and two (0.78%) DEPs in the IC area were common to DEGs in the C and IC areas between pregnant (after being bred to rams) and cyclic ewes [8]. The relatively few overlapping DEPs implied that the expression patterns characterizing early pregnancy loss are considerably distinct from those characterizing establishment of pregnancy.

An notable observation is that in both the C and IC areas, more DEPs showed significant downregulation in non-pregnant ewes. Considering the fact that the expression of a large number of genes are activated by pregnancy signals derived from the conceptus, as well as the fact that all embryos transferred in the two groups were unified and well-developed blastocysts, the lower protein abundance in the non-pregnant endometrium may be attributed to an insufficient maternal response to the pregnancy signals.

### IFN signaling and immune response

IFNT (IFN tau) secreted by the conceptus trophectoderm is the primary signal for maternal recognition of pregnancy in ewes between Day 10 and Day 20 of pregnancy, with maximum production by the trophoblast on Day 15 and Day 16 [31,32]. Although IFNT was originally described based on its ability to mediate pregnancy recognition, immunological responses and prevent viral infection in various cell types, its potential role in embryo implantation is still not fully understood [33]. Pregnancy is characterized by mild systemic immunosuppression and inflammation, which is essential for the maternal-conceptus dialog during pregnancy recognition [34]. Therefore, understanding IFNT-regulated immune modifications in the endometrium would help to discern the underlying mechanism of failed early pregnancy.

**Figure 5:**
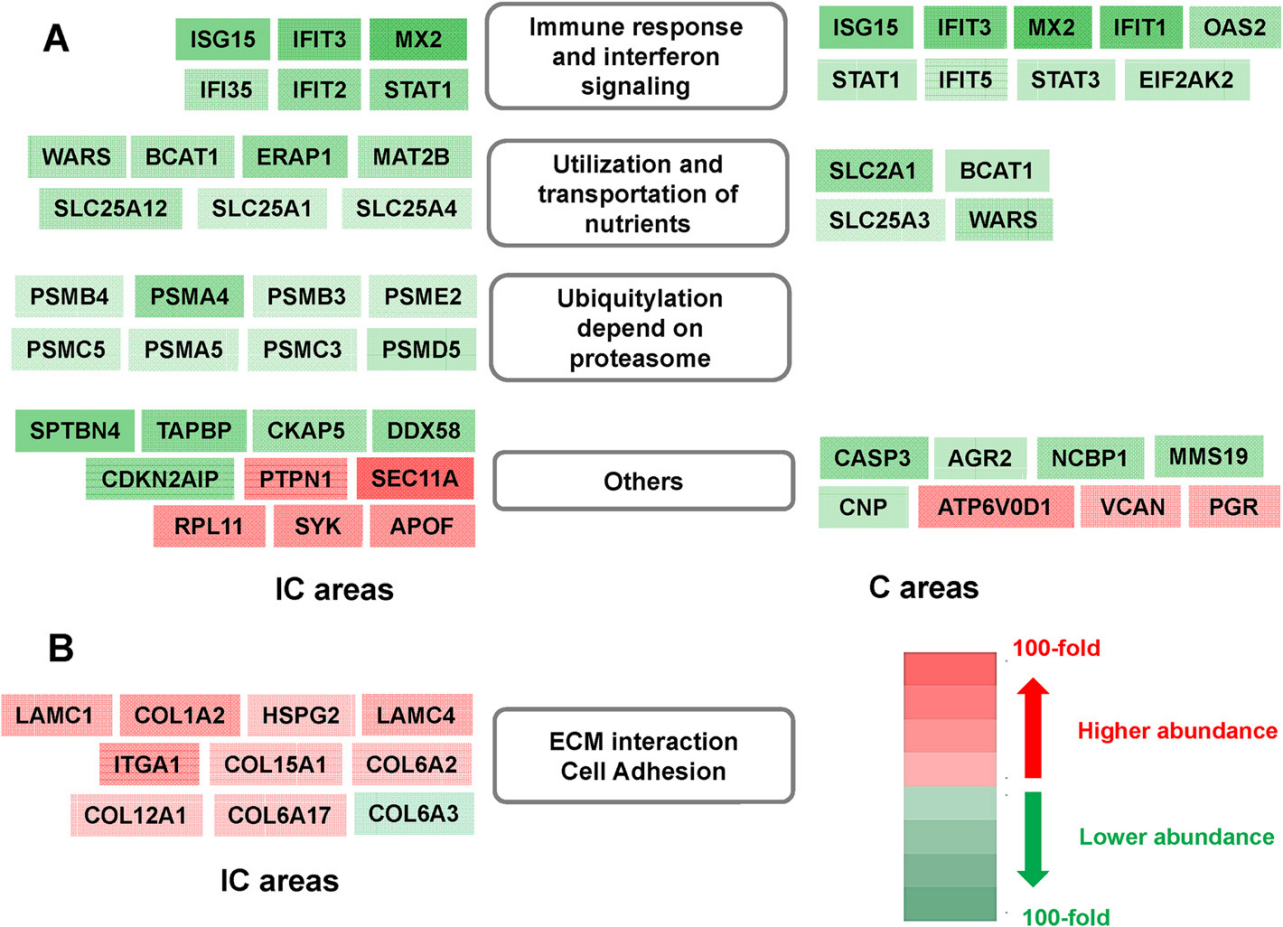
Schematic representation of processes and corresponding proteins with putative functions in the endometrium associated with failed early pregnancy. **(A)** The endometrial regions of pregnant and non-pregnant ewes were subdivided into C and IC areas. Each DEP (pregnant *vs*. non-pregnant) is represented as a box within the major biological process to which it contributes. Proteins in red boxes were more abundant in the endometrium of non-pregnant ewes than those in pregnant ewes. Proteins in green boxes were present at lower abundance in the endometrium of non-pregnant ewes than those in pregnant ewes. **(B)**. Representation of DEPs (IC *vs*. C areas in the pregnant endometrium). Proteins in red boxes were more abundant in the IC area than that in the C area. Proteins in green boxes were less abundant in the IC area than that in the C area. Gene symbols represent the proteins.

In the present study, many of the significantly downregulated proteins in the endometrium of non-pregnant ewes are known as ISG (IFN-stimulated gene) proteins (Figure 5A). Among these, the abundances of MX2, ISG15 and IFIT3 were altered to the greatest extent. It should be mentioned that the corresponding genes, especially MX2 (659-fold) and ISG15 (2390-fold-fold), also showed dramatically induced upregulation in related transcriptome studies concerning pregnancy establishment [7]. Previous reports indicated that MX is induced by IFNT during implantation in pregnant ruminants [35]. The peak expression of MX occurs on Day 17 of pregnancy in ewes, perhaps to offset the immunomodulatory effects of progesterone [36]. ISG15 was also suggested to play an important role in IFNT signaling [37]. Enhanced expression of ISG15 in the endometrium occurs during early pregnancy in cows [38] and sheep [39]. ISG15-induced expression of E-cadherin might play role in escape of tumors from immune cells [40], implying that E-cadherin may participate in preventing rejection of the conceptus by the maternal immune system. In addition, ISG15 participates in the differentiation and proliferation of NK (natural killer) cells and activation of NK cell-derived lymphokine-activated killer cells [41]. These results suggest that ISG15 may be involved in the migration of specialized immune cells and the induction of vascular remodeling and angiogenesis; however, it does not induce a massive immune response that would be detrimental to the implanting blastocysts. The importance of ISG15 in early pregnancy may be appreciated by the fact that up to 50% of embryos die in ISG15^−/−^ mice [42]. In the present study, the IFIT protein also showed a dramatic decrease in the endometrium of the non-pregnant ewes. Xiao *et al.* described IFIT3 as a key mediator of anti-proliferative activity of IFNs [43]. Other ISGs were also obviously deficient in the endometrium of non-pregnant ewes in our study, such as EIF2AK2 (eukaryotic translation initiation factor 2-alpha kinase 2), OAS2, STAT1 and STAT3 (signal transducer and activator of transcription 3), which may play important roles in IFN-mediated cell signaling and immune responses.

During early pregnancy, many proteins involved in immune responses may be responsible for inducing tolerance to the embryo to protect the uterus against infection during a time of local immune suppression [23]. As well-defined ISGs, the deficiency of these proteins may reflect the insufficient response of the endometrium to IFNT, suggesting that an aberrant response of the endometrium to IFNT, as well as subsequent suboptimal endometrial modulation, may be an important characteristics to failed early pregnancy.

### Nutrient transport and utilization

Transport and utilization of uterine secretions (termed histotroph) are critical for growth of the trophectoderm, as well as survival and elongation of the conceptus. The histotroph provide major nutrients, such as an energy source and amino acids, in embryonic development. Glucose and glutamine, as major metabolic fuels for the conceptus, can regulate the proliferation and function of trophoblast cells [44]. Amino acids also play significant roles in conceptus development by influencing trophectoderm proliferation, differentiation, migration and implantation [45,46]. According to the assigned GO terms and KEGG pathways, DEPs between pregnant and non-pregnant groups were enriched in functions related to utilization and transport of energy and amino acids (Figure 2 and Table 1).

Gao *et al.* previously reported that some members of the SLC protein family have important roles in the transport of glucose [47] and amino acids during the peri-implantation period of pregnancy [48]. In our study, a dramatic decrease (11.8-fold) in SLC2A1 was observed in the C area of the non-pregnant ewes compared with the pregnant ewes. In previous studies, a significant increase of SLC2A1 was been reported in pregnant bovines compared with cyclic bovines, implying the participation of this glucose transporter in the establishment of pregnancy [7]. Our results indicated that deficiency of SLC2A1 may lead to aberrant transport of glucose in non-pregnant ewes. We also detected a significant downregulation of other members of the SLC family, including SLC25A1 [solute carrier family 25 (mitochondrial carrier; phosphate carrier), member 1] and SLC25A12 [solute carrier family 25 (mitochondrial carrier, Aralar), member 12] in the endometrium of the non-pregnant ewes (Figure 5A). SLC25 transporters, a large family of nuclear-encoded membrane-embedded proteins that promote solute transport across the inner mitochondrial membrane, have been implicated as being responsible for the transport and utilization of glutamate [49]. Decreases in the levels of these members of the SLC family may affect nutrient transport and utilization in the endometrium. Additionally, we found that some proteins that participate in amino acid metabolism, such as ERAP1, MAT2B, WARS (tryptophanyl-tRNA synthetase) and BCAT1 (branched-chain-amino-acid aminotransferase), were more abundant in the endometrium of the pregnant ewes (Figure 5A).

Many proteins involved in the transport and utilization of energy substrates and amino acids were identified to have significant differences in abundance between pregnant and non-pregnant ewes in our study. Consistent with previous studies [50,51], our results indicated that aberrant expression of glucose and amino acid transporters in the endometrium may contribute largely to the impaired nutritional utilization, which is highly associated with compromised embryonic development and survival. This finding suggests that, for successful implantation, the conceptus must stimulate endometrial metabolism and, in particular, increase the abundance of selected nutrients to improve uterine receptivity.

### Proteasome-dependent proteolysis

The proteasome is an ATP-dependent, multi-subunit, multi-catalytic protease complex that is responsible for recognizing and degrading ubiquitinated proteins in the majority of non-lysosomal degradation events in eukaryotic cells, including the degradation of misfolded, damaged and other regulatory proteins [52,53]. Ubiquitination-related proteins present in cells of the pregnant uterus in many species may have key roles in endometrial modification and placental development during pregnancy [37,54,55]. As far as we know, few studies have reported a role for the proteasome in the ovine endometrium during early pregnancy. Our results imply that the proteasome may participate in the preparation of the endometrium for conceptus implantation (Table 1).

As an indispensable component of the ubiquitin-proteasome system (UPS), the proteasome participates in many fundamental biological processes, including the extensive tissue remodeling of the uterus and conceptus during pregnancy [56]. In mice, inhibition of proteasome subunits can significantly decrease the abundance of proteins responsible for degradation of the ECM, which is required during tissue remodeling [57]. During early pregnancy, proteasome subunits may affect secretions by uterine glands, as well as generation of MHC class I molecules and antigenic peptides that increase the immune tolerance of the maternal immune system to conceptusin the rhesus monkey [58]. In the present study, various members of the proteasome family subunits, such as PSMA4, PSMA5 and PSME2, were significantly downregulated in non-pregnant ewes (Figure 5A), suggesting that depressed proteasome-dependent proteolysis may be involved in early pregnancy failure. Although no proteins associated with UPS in our study were common to previous related studies, other proteasome family subunits, such as PSMB8 and PSMB9, were identified as increased in the endometrium during implantation in cows.

### ECM-receptor interaction and cell adhesion

In pregnant ewes, it is noteworthy that the abundance of endometrial proteins involved in the “ECM-receptor interaction” (KEGG pathway) increased dramatically in the IC area during the peri-implantation period Figure 5B). In addition, other proteins associated with “focal adhesion assembly” (KEGG pathway) were also highly expressed in the IC area of pregnant ewes (Figure 5B). By contrast, no similar proteomic patterns were detected in non-pregnant ewes.

Cell-to-cell communication is essential in many physiological processes and are particularly crucial during embryonic development of multicellular organisms [59]. From the very first stage of embryogenesis, cells establish contact with the extracellularmatrix (ECM), and this interaction would influence directly or indirectly numerous aspects of cell behavior, cell migration, cell growth, cell survival, cell proliferation, angiogenesis, invasion [60]. During the peri-implantation period of pregnancy, ECM interactions and cell adhesion-dependent biological processes are very important in the endometrial IC area. Previous reports indicated that endometrial glands in the IC area undergo extensive hyperplasia and hypertrophy during pregnancy, presumably to meet the increasing demands of the developing conceptus for uterine histotroph [22,61,62]. This process requires site-specific alterations in cell proliferation and ECM remodeling, as well as paracrine cell-cell and cell-ECM interactions that support the actions of specific hormones and growth factors [63]. Integrins bind ECM molecules to transduce cell signals and mediate adhesion, and reorganize the cytoskeleton to stabilize adhesion and cell hypertrophy, which are crucial for implantation and placentation in all mammals [64].

The observed aberrant expression patterns of ECM molecules between the IC and C areas of the endometrium, suggests that ECM-receptor Interaction is essential for endometrial functional differentiation, of which deficiency may result in failed maternal–fetal cross-talk. In addition, traditional studies on ECM and cell adhesion have generally focused on the C area, which is the site for attachment and adhesion of the trophoblast leading to formation of a placentome. However, consistent with the viewpoints of Bazer [22] and Gray [63], our results suggested that ECM interactions and cell adhesion-dependent biological processes also have very important roles in the IC area during the peri-implantation period of pregnancy, which has not been reported previously.

## Conclusions

In conclusion, LC-ESI-/MS-MS technology allowed us to develop the first proteomic map in the sheep endometrium and to obtain a distinctive proteomic profile for the endometrium of ewes experiencing early pregnancy failure. Many dysregulated proteins were identified in the endometrium of non-pregnant ewes, some of which had been reported to be involved in establishment of pregnancy, while the roles of other newly identified proteins in early pregnancy failure remain to be further investigated. Based on the functional enrichment analysis of DEPs by GO, KEGG and network categories, a general conclusion can be drawn that aberrant nutrient transport and utilization, abnormal immune responses, as well as aberrant proteasome-dependent proteolysis in the endometrium are tightly associated with early pregnancy failure in sheep (Figure 5A). In addition, here we displayed a interesting novel finding. The highly abundant protein related to “ECM-receptor interaction” and “focal adhesion assembly” suggests that cell adhesion is also presented in IC areas, which may be responsible for the successful pregnancy establishement. The results of our research provide a comprehensive and detailed reference for further studies to advance the understanding of the mechanism(s) associated with early pregnancy failure. It should be mentioned that few proteomic data of sheep could be validated in previous related studies, and the validation of our proteomic data by western blotting were not provided because of the non-availability of effective antibodies. However, this did not compromise the significance of our proteomic data as a reference for exploring the underlying mechanism of endometrium-associated early pregnancy failure.

## Additional files

Additional file 1: Table S2-S5. Lists of proteins identified in each sample. **Table S6-S9.** Lists of DEPs in each comparison. **Table S10.** Total peptides identified in each sample. Additional file 2: Table S1. Summary of Pearson’s correlation coefficients (R) between technical replicates in each pool of the different groups. Additional file 3: Figure S1. Pearson correlation coefficients (PCCs) of protein levels between technique replicates in each pool of the different groups. The reliability of protein quantitation between the technical replicates was evaluated by the PCCs. Additional file 4: Figure S2. Hierarchical clustering analyses of C areas (A) and IC areas (B) based on protein expression patterns of all technical and biological replicates in the different groups. Different colors represent normalized protein abundances (LFQ-intensities): red indicates high abundance and green denotes low abundance. Additional file 5: Figure S3. Distribution of DEPs (pregnant *vs*. non-pregnant) with different fold changes (FC) in C and IC areas. Red and blue bars show proteins that were significantly upregulated and downregulated, respectively, in pregnant and non-pregnant ewes (P < 0.05). Dark and pale colors correspond to the different fold changes. Additional file 6: Figure S4. Interactive network analysis of DEPs between pregnant and non-pregnant ewes in the C area. Proteins enclosed in color-coded outlines are mainly involved in carbohydrate metabolism (red) and interferon signaling (green). Additional file 7: Figure S5. Gene Ontology “biological process” classifications of DEPs (C *vs*. IC) in the pregnant (A) and non-pregnant (B) ewes. Additional file 8: Figure S6. Venn diagrams for retrospective analysis of DEPs in the present study with previously published related studies [7,8]. (A) Comparison of candidate proteins associated with failed pregnancy with gens responsible for pregnancy establishment in C and IC areas respectively [7]. (B) Comparison of candidate proteins associated with failed pregnancy in C and IC areas with endometrial genes responsible for pregnancy establishment [8].

## References

[1] Bazer FW, Wu G, Johnson GA, Kim J, Song G (2011). Uterine histotroph and conceptus development: select nutrients and secreted phosphoprotein 1 affect mechanistic target of rapamycin cell signaling in ewes. Biol Reprod.

[2] Kwak‐Kim J, Park JC, Ahn HK, Kim JW, Gilman‐Sachs A (2010). Review article: immunological modes of pregnancy loss. Am J Reprod Immunol.

[3] Dixon A, Knights M, Winkler J, Marsh D, Pate J, Wilson M, Dailey R, Seidel G, Inskeep E (2007). Patterns of late embryonic and fetal mortality and association with several factors in sheep. J Anim Sci.

[4] Lee KY, DeMayo FJ (2004). Animal models of implantation. Reproduction.

[5] Mansouri-Attia N, Sandra O, Aubert J, Degrelle S, Everts RE, Giraud-Delville C, Heyman Y, Galio L, Hue I, Yang X (2009). Endometrium as an early sensor of in vitro embryo manipulation technologies. Proc Natl Acad Sci.

[6] Salker M, Teklenburg G, Molokhia M, Lavery S, Trew G, Aojanepong T, Mardon HJ, Lokugamage AU, Rai R, Landles C (2010). Natural selection of human embryos: impaired decidualization of endometrium disables embryo-maternal interactions and causes recurrent pregnancy loss. PLoS One.

[7] Mansouri-Attia N, Aubert J, Reinaud P, Giraud-Delville C, Taghouti G, Galio L, Everts RE, Degrelle S, Richard C, Hue I (2009). Gene expression profiles of bovine caruncular and intercaruncular endometrium at implantation. Physiol Genomics.

[8] Gray CA, Abbey CA, Beremand PD, Choi Y, Farmer JL, Adelson DL, Thomas TL, Bazer FW, Spencer TE (2006). Identification of endometrial genes regulated by early pregnancy, progesterone, and interferon tau in the ovine uterus. Biol Reprod.

[9] Patel BG, Lessey BA (2011). Clinical assessment and management of the endometrium in recurrent early pregnancy loss. In Seminars in reproductive medicine. Thieme Medical Publishers.

[10] Niklaus AL, Pollard JW (2006). Mining the mouse transcriptome of receptive endometrium reveals distinct molecular signatures for the luminal and glandular epithelium. Endocrinology.

[11] Østrup E, Bauersachs S, Blum H, Wolf E, Hyttel P (2010). Differential endometrial gene expression in pregnant and nonpregnant sows. Biol Reprod.

[12] Klein C, Scoggin KE, Ealy AD, Troedsson MH (2010). Transcriptional profiling of equine endometrium during the time of maternal recognition of pregnancy. Biol Reprod.

[13] Walker CG, Meier S, Littlejohn MD, Lehnert K, Roche JR, Mitchell MD (2010). Modulation of the maternal immune system by the pre-implantation embryo. BMC Genomics.

[14] Bauersachs S, Ulbrich SE, Gross K, Schmidt SE, Meyer HH, Wenigerkind H, Vermehren M, Sinowatz F, Blum H, Wolf E (2006). Embryo-induced transcriptome changes in bovine endometrium reveal species-specific and common molecular markers of uterine receptivity. Reproduction.

[15] Klein C, Bauersachs S, Ulbrich SE, Einspanier R, Meyer HH, Schmidt SE, Reichenbach HD, Vermehren M, Sinowatz F, Blum H, Wolf E (2006). Monozygotic twin model reveals novel embryo-induced transcriptome changes of bovine endometrium in the preattachment period. Biol Reprod.

[16] Wan PC, Bao ZJ, Wu Y, Yang L, Hao ZD, Yang YL, Shi GQ, Liu Y, Zeng SM (2011). alphav beta3 Integrin may participate in conceptus attachment by regulating morphologic changes in the endometrium during peri-implantation in ovine. Reprod Domest Anim.

[17] Spencer TE, Bazer FW (2004). Conceptus signals for establishment and maintenance of pregnancy. Reprod Biol Endocrinol.

[18] Gray CA, Adelson DL, Bazer FW, Burghardt RC, Meeusen EN, Spencer TE (2004). Discovery and characterization of an epithelial-specific galectin in the endometrium that forms crystals in the trophectoderm. Proc Natl Acad Sci U S A.

[19] Song G, Satterfield MC, Kim J, Bazer FW, Spencer TE (2008). Gastrin-releasing peptide (GRP) in the ovine uterus: regulation by interferon tau and progesterone. Biol Reprod.

[20] Fleming JA, Song G, Choi Y, Spencer TE, Bazer FW (2009). Interferon regulatory factor 6 (IRF6) is expressed in the ovine uterus and functions as a transcriptional activator. Mol Cell Endocrinol.

[21] Choi Y, Johnson GA, Burghardt RC, Berghman LR, Joyce MM, Taylor KM, Stewart MD, Bazer FW, Spencer TE (2001). Interferon regulatory factor-two restricts expression of interferon-stimulated genes to the endometrial stroma and glandular epithelium of the ovine uterus. Biol Reprod.

[22] Bazer FW (1975). Uterine protein secretions: relationship to development of the conceptus. J Anim Sci.

[23] Walker CG, Meier S, Littlejohn MD, Lehnert K, Roche JR, Mitchell MD (2010). Modulation of the maternal immune system by the pre-implantation embryo. BMC Genomics.

[24] Satterfield MC, Song G, Kochan KJ, Riggs PK, Simmons RM, Elsik CG, Adelson DL, Bazer FW, Zhou H, Spencer TE (2009). Discovery of candidate genes and pathways in the endometrium regulating ovine blastocyst growth and conceptus elongation. Physiol Genomics.

[25] Cox J, Mann M (2008). MaxQuant enables high peptide identification rates, individualized ppb-range mass accuracies and proteome-wide protein quantification. Nat Biotechnol.

[26] Waanders LF, Chwalek K, Monetti M, Kumar C, Lammert E, Mann M (2009). Quantitative proteomic analysis of single pancreatic islets. Proc Natl Acad Sci.

[27] Wang G, Wu WW, Zeng W, Chou C-L, Shen R-F (2006). Label-free protein quantification using LC-coupled ion trap or FT mass spectrometry: Reproducibility, linearity, and application with complex proteomes. J Proteome Res.

[28] Huang DW, Sherman BT, Lempicki RA (2008). Systematic and integrative analysis of large gene lists using DAVID bioinformatics resources. Nat Protoc.

[29] Huang DW, Sherman BT, Tan Q, Kir J, Liu D, Bryant D, Guo Y, Stephens R, Baseler MW, Lane HC (2007). DAVID Bioinformatics Resources: expanded annotation database and novel algorithms to better extract biology from large gene lists. Nucleic Acids Res.

[30] Geiger T, Wehner A, Schaab C, Cox J, Mann M (2012). Comparative proteomic analysis of eleven common cell lines reveals ubiquitous but varying expression of most proteins. Mol Cell Proteomics.

[31] Farin C, Imakawa K, Roberts R (1989). In situ localization of mRNA for the interferon, ovine trophoblast protein-1, during early embryonic development of the sheep. Mol Endocrinol.

[32] Ashworth C, Bazer F (1989). Changes in ovine conceptus and endometrial function following asynchronous embryo transfer or administration of progesterone. Biol Reprod.

[33] Ealy AD, Yang QE (2009). REVIEW ARTICLE: Control of Interferon‐Tau Expression During Early Pregnancy in Ruminants. Am J Reprod Immunol.

[34] Warning JC, McCracken SA, Morris JM (2011). A balancing act: mechanisms by which the fetus avoids rejection by the maternal immune system. Reproduction.

[35] Klein C, Bauersachs S, Ulbrich SE, Einspanier R, Meyer HH, Schmidt SE, Reichenbach H-D, Vermehren M, Sinowatz F, Blum H (2006). Monozygotic twin model reveals novel embryo-induced transcriptome changes of bovine endometrium in the preattachment period. Biol Reprod.

[36] Ott TL, Yin J, Wiley AA, Kim H-T, Gerami-Naini B, Spencer TE, Bartol FF, Burghardt RC, Bazer FW (1998). Effects of the estrous cycle and early pregnancy on uterine expression of Mx protein in sheep (Ovis aries). Biol Reprod.

[37] Johnson GA, Austin KJ, Van Kirk EA, Hansen TR (1998). Pregnancy and interferon-tau induce conjugation of bovine ubiquitin cross-reactive protein to cytosolic uterine proteins. Biol Reprod.

[38] Johnson GA, Austin KJ, Collins AM, Murdoch WJ, Hansen TR (1999). Endometrial ISG17 mRNA and a related mRNA are induced by interferon-tau and localized to glandular epithelial and stromal cells from pregnant cows. Endocrine.

[39] Nighswonger A, Austin K, Ealy A, Han C, Hansen T (2000). Rapid communication: the ovine cDNA encoding interferon-stimulated gene product 17 (ISG17). J Anim Sci.

[40] Padovan E, Terracciano L, Certa U, Jacobs B, Reschner A, Bolli M, Spagnoli GC, Borden EC, Heberer M (2002). Interferon stimulated gene 15 constitutively produced by melanoma cells induces e-cadherin expression on human dendritic cells. Cancer Res.

[41] D’Cunha J, Knight E, Haas AL, Truitt RL, Borden EC (1996). Immunoregulatory properties of ISG15, an interferon-induced cytokine. Proc Natl Acad Sci.

[42] Ashley RL, Henkes LE, Bouma GJ, Pru JK, Hansen TR (2010). Deletion of the Isg15 gene results in up-regulation of decidual cell survival genes and down-regulation of adhesion genes: implication for regulation by IL-1β. Endocrinology.

[43] Xiao S, Li D, Zhu H-Q, Song M-G, Pan X-R, Jia P-M, Peng L-L, Dou A-X, Chen G-Q, Chen S-J (2006). RIG-G as a key mediator of the antiproliferative activity of interferon-related pathways through enhancing p21 and p27 proteins. Proc Natl Acad Sci.

[44] Petters R, Johnson B, Reed M, Archibong A (1990). Glucose, glutamine and inorganic phosphate in early development of the pig embryo in vitro. J Reprod Fertil.

[45] Martin PM, Sutherland AE (2001). Exogenous amino acids regulate trophectoderm differentiation in the mouse blastocyst through an mTOR-dependent pathway. Dev Biol.

[46] Martin PM, Sutherland AE, Van Winkle LJ (2003). Amino acid transport regulates blastocyst implantation. Biol Reprod.

[47] Gao H, Wu G, Spencer TE, Johnson GA, Bazer FW (2009). Select nutrients in the ovine uterine lumen. II. Glucose transporters in the uterus and peri-implantation conceptuses. Biol Reprod.

[48] Gao H, Wu G, Spencer TE, Johnson GA, Bazer FW (2009). Select nutrients in the ovine uterine lumen. III. Cationic amino acid transporters in the ovine uterus and peri-implantation conceptuses. Biol Reprod.

[49] Palmieri F (2008). Diseases caused by defects of mitochondrial carriers: a review. Biochim Biophys Acta.

[50] Frolova AI, Moley KH (2011). Quantitative analysis of glucose transporter mRNAs in endometrial stromal cells reveals critical role of GLUT1 in uterine receptivity. Endocrinology.

[51] Groebner AE, Rubio-Aliaga I, Schulke K, Reichenbach HD, Daniel H, Wolf E, Meyer HH, Ulbrich SE (2011). Increase of essential amino acids in the bovine uterine lumen during preimplantation development. Reproduction.

[52] Coux O, Tanaka K, Goldberg AL (1996). Structure and functions of the 20S and 26S proteasomes. Annu Rev Biochem.

[53] Voges D, Zwickl P, Baumeister W (1999). The 26S proteasome: a molecular machine designed for controlled proteolysis. Annu Rev Biochem.

[54] Bebington C, Doherty F, Fleming S (2000). Ubiquitin and ubiquitin-protein conjugates are present in human cytotrophoblast throughout gestation. Early Pregnancy.

[55] Johnson GA, Spencer TE, Hansen TR, Austin KJ, Burghardt RC, Bazer FW (1999). Expression of the interferon tau inducible ubiquitin cross-reactive protein in the ovine uterus. Biol Reprod.

[56] Bebington C, Doherty F, Fleming S (2001). The possible biological and reproductive functions of ubiquitin. Hum Reprod Update.

[57] Wang HM, Zhang X, Qian D, Lin HY, Li QL, Liu DL, Liu GY, Yu XD, Zhu C (2004). Effect of ubiquitin-proteasome pathway on mouse blastocyst implantation and expression of matrix metalloproteinases-2 and-9. Biol Reprod.

[58] Wang H-X, Wang H-M, Li Q-L, Lin H-Y, Qian D, Zhu C (2004). Expression of proteasome subunits low molecular mass polypeptide (LMP) 2 and LMP7 in the endometrium and placenta of rhesus monkey (Macaca mulatta) during early pregnancy. Biol Reprod.

[59] Hynes RO (1992). Integrins: versatility, modulation, and signaling in cell adhesion. Cell.

[60] Reddy K, Mangale SS (2003). Integrin receptors: the dynamic modulators of endometrial function. Tissue Cell.

[61] Wimsatt WA (1950). New histological observations on the placenta of the sheep. Am J Anat.

[62] Stewart MD, Johnson GA, Gray CA, Burghardt RC, Schuler LA, Joyce MM, Bazer FW, Spencer TE (2000). Prolactin receptor and uterine milk protein expression in the ovine endometrium during the estrous cycle and pregnancy. Biol Reprod.

[63] Gray CA, Bartol FF, Tarleton BJ, Wiley AA, Johnson GA, Bazer FW, Spencer TE (2001). Developmental biology of uterine glands. Biol Reprod.

[64] Burghardt RC, Burghardt JR, Taylor JD, Reeder AT, Nguen BT, Spencer TE, Bayless KJ, Johnson GA (2009). Enhanced focal adhesion assembly reflects increased mechanosensation and mechanotransduction at maternal–conceptus interface and uterine wall during ovine pregnancy. Reproduction.

