# Supplementary material for: Comparative analysis between endometrial proteomes of pregnant and non-pregnant ewes during the peri-implantation period
Source: J Anim Sci Biotechnol. 2015 Apr 25;6(1):18. doi: 10.1186/s40104-015-0017-0 (PMC4447021; doi:10.1186/s40104-015-0017-0)
Supplement: Additional file 2: Table S1. — Summary of Pearson’s correlation coefficients (R) between technical replicates in each pool of the different groups. [file 40104_2015_17_MOESM2_ESM.docx]

| **Group** | **Biological replicates** | **Technical replicates** | **R(peptide level)** | **R(protein level)** |
| --- | --- | --- | --- | --- |
| pregnant_C | Pool 1 | rep1_rep2 | 0.9524 | 0.9833 |
|  | Pool 2 | rep1_rep2 | 0.9613 | 0.9861 |
|  | Pool 3 | rep1_rep2 | 0.9465 | 0.9777 |
| pregnant_IC | Pool 1 | rep1_rep2 | 0.9339 | 0.9923 |
|  | Pool 2 | rep1_rep2 | 0.9119 | 0.9967 |
|  | Pool 3 | rep1_rep2 | 0.9614 | 0.9866 |
| non-pregnant_C | Pool 1 | rep1_rep2 | 0.9466 | 0.9850 |
|  | Pool 2 | rep1_rep2 | 0.9772 | 0.9955 |
|  | Pool 3 | rep1_rep2 | 0.9580 | 0.9907 |
| non-pregnant_IC | Pool 1 | rep1_rep2 | 0.9721 | 0.9838 |
|  | Pool 2 | rep1_rep2 | 0.9506 | 0.9968 |
|  | Pool 3 | rep1_rep2 | 0.9737 | 0.9881 |
|  |  | Average R | 0.9492 | 0.9886 |
|  |  | Range of R values | 0.9254-0.9772 | 0.9777-0.9968 |

**Supplemental Table S1:** Summary of Pearson’s correlation coefficient (R) between technical replicates in each pool of different groups.
