# Supplementary figures and images for: Comparative analysis between endometrial proteomes of pregnant and non-pregnant ewes during the peri-implantation period
Source: J Anim Sci Biotechnol. 2015 Apr 25;6(1):18. doi: 10.1186/s40104-015-0017-0 (PMC4447021; doi:10.1186/s40104-015-0017-0)

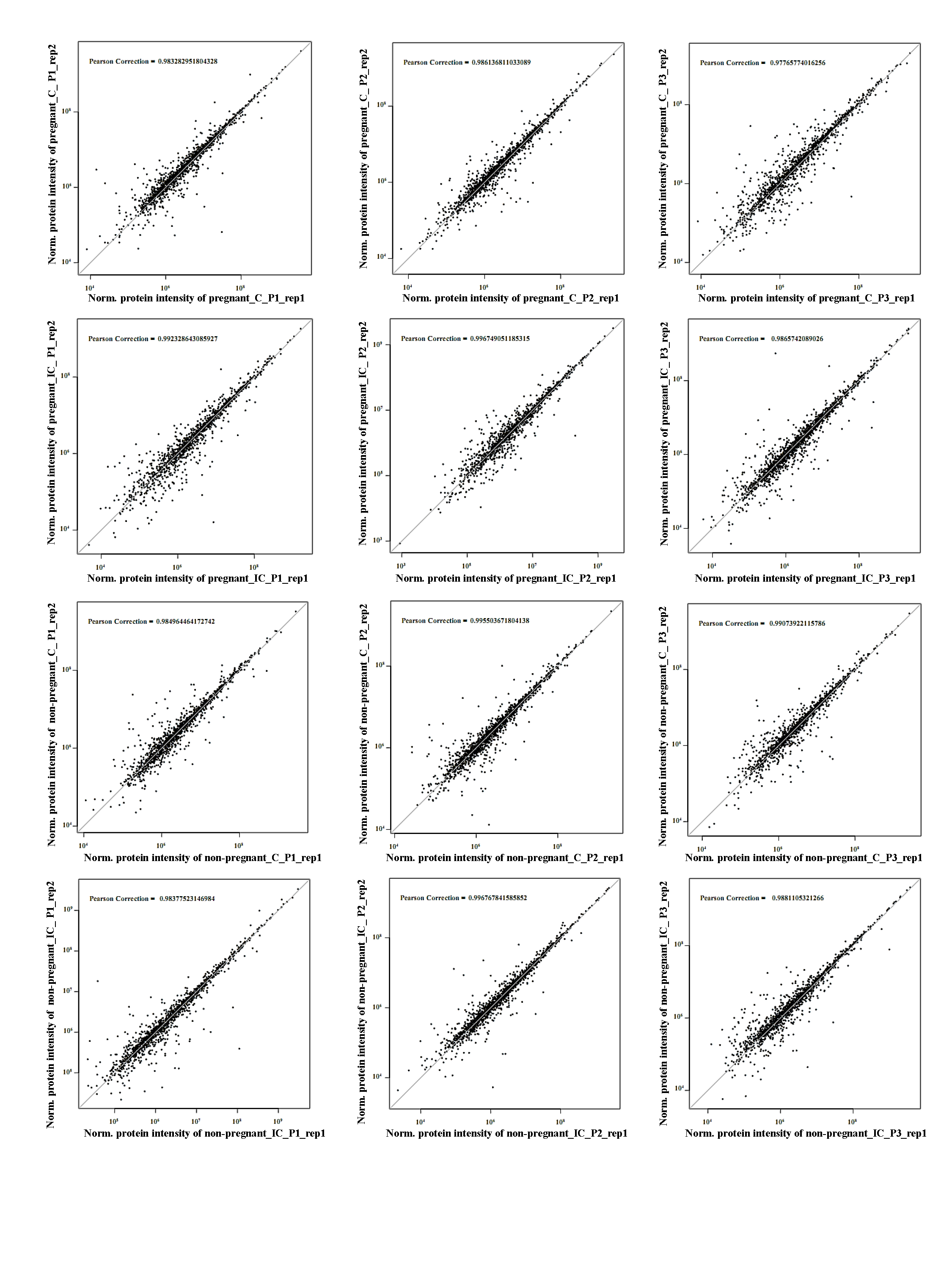

Supplement: Additional file 3: Figure S1. — Pearson correlation coefficients (PCCs) of protein levels between technique replicates in each pool of the different groups. The reliability of protein quantitation between the technical replicates was evaluated by the PCCs. [file 40104_2015_17_MOESM3_ESM.tiff]

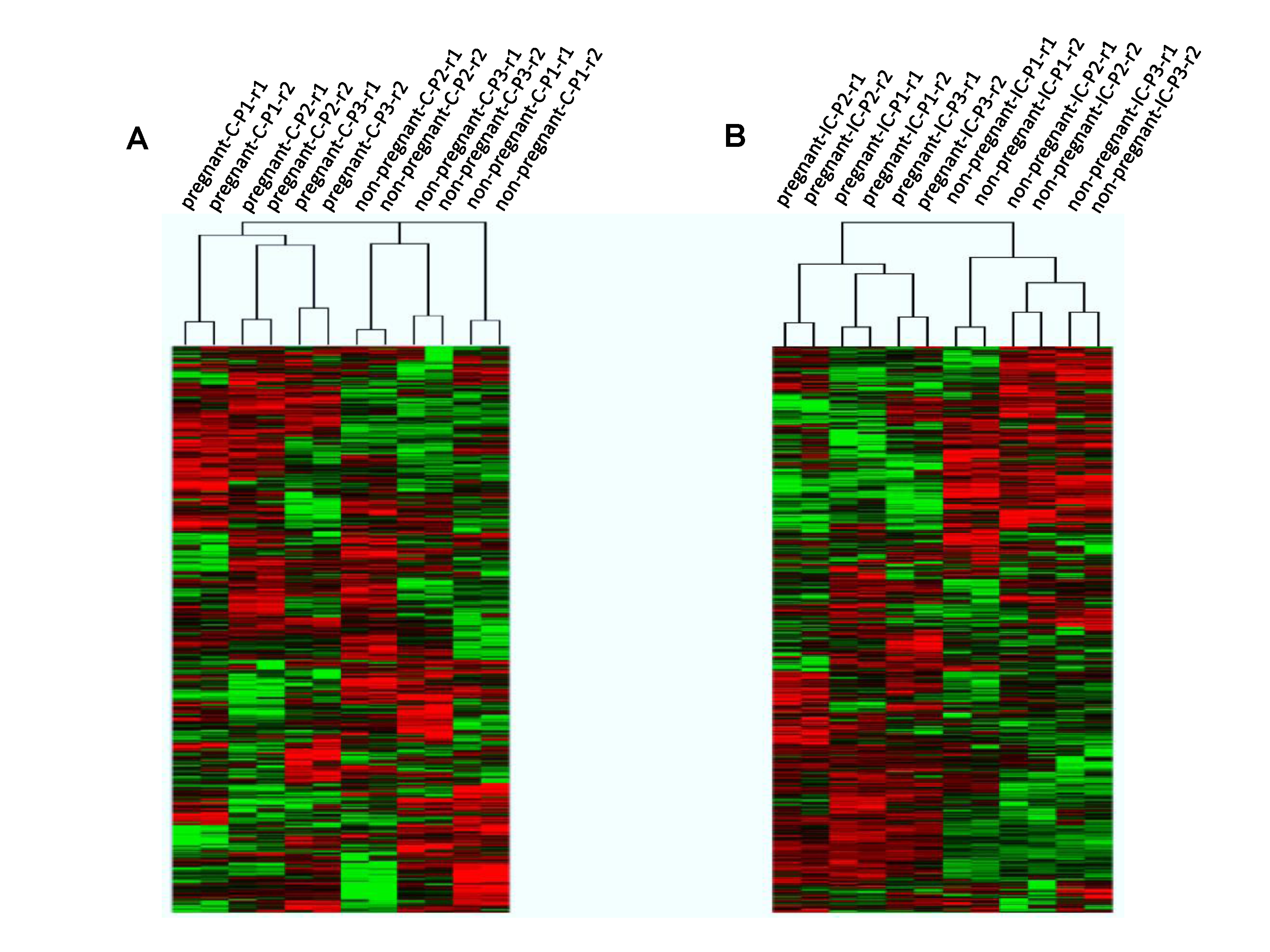

Supplement: Additional file 4: Figure S2. — Hierarchical clustering analyses of C areas (A) and IC areas (B) based on protein expression patterns of all technical and biological replicates in the different groups. Different colors represent normalized protein abundances (LFQ-intensities): red indicates high abundance and green denotes low abundance. [file 40104_2015_17_MOESM4_ESM.tiff]

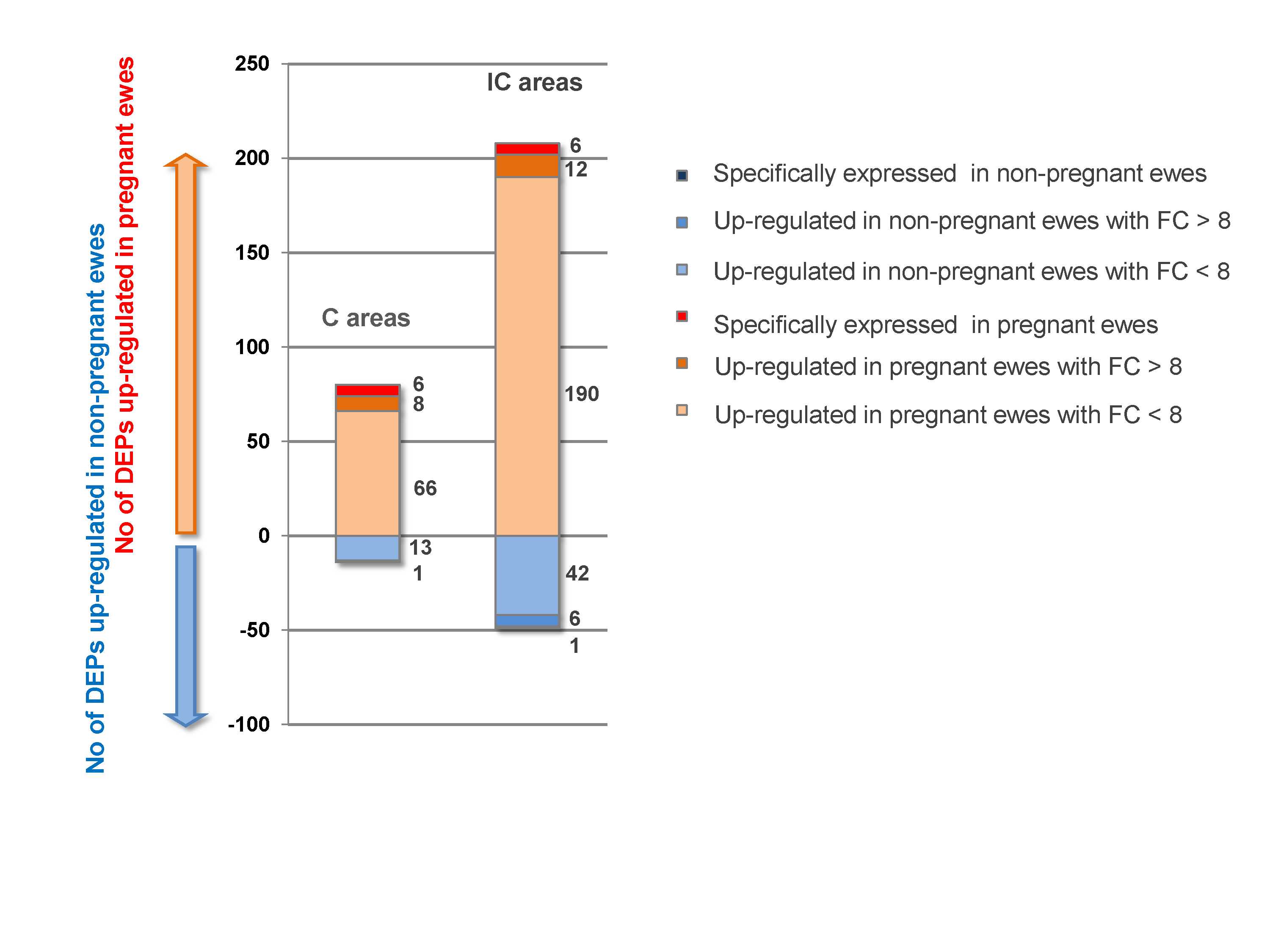

Supplement: Additional file 5: Figure S3. — Distribution of DEPs (pregnant vs. non-pregnant) with different fold changes (FC) in C and IC areas. Red and blue bars show proteins that were significantly upregulated and downregulated, respectively, in pregnant and non-pregnant ewes (P < 0.05). Dark and pale colors correspond to the different fold changes. [file 40104_2015_17_MOESM5_ESM.tiff]

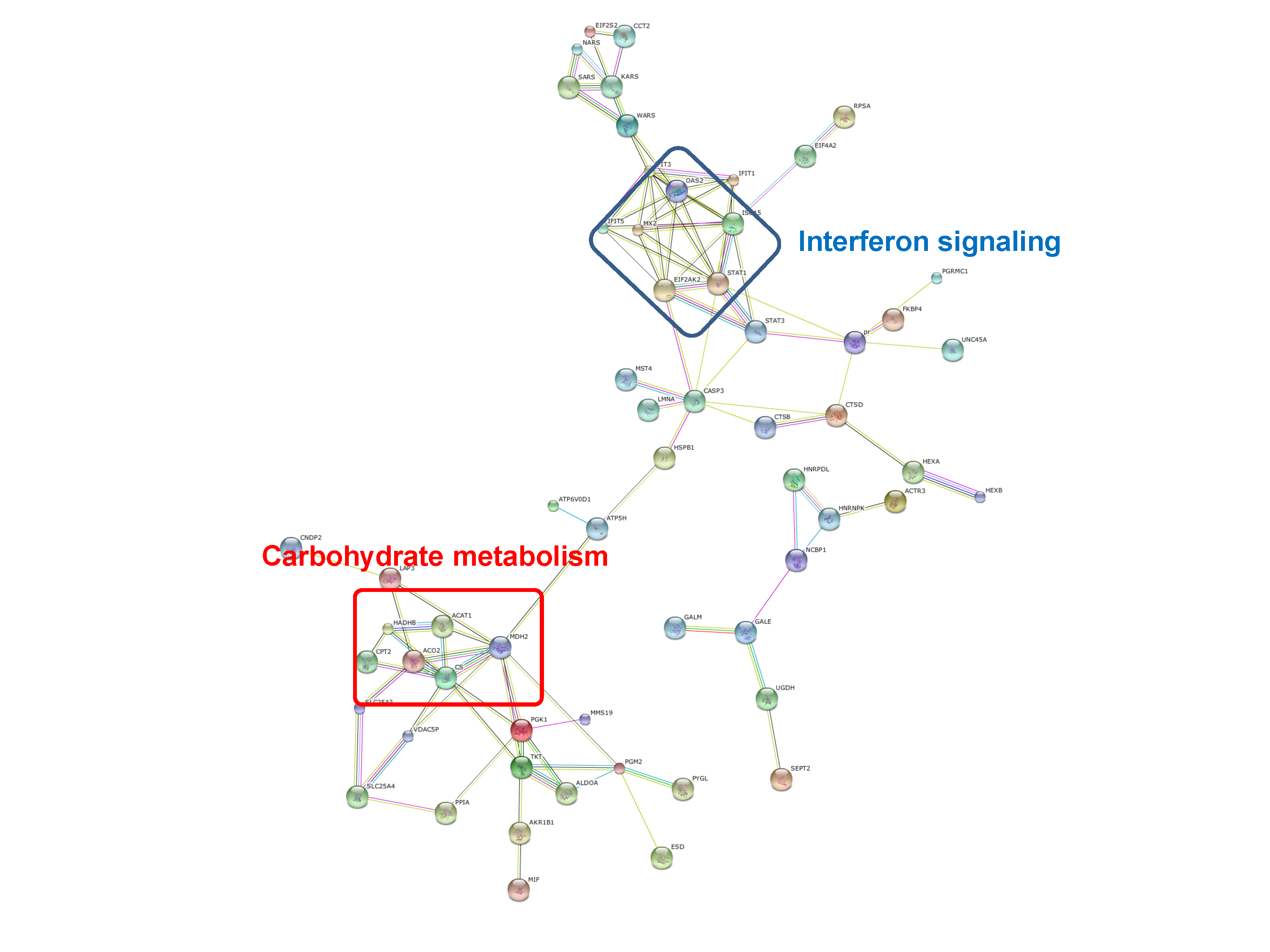

Supplement: Additional file 6: Figure S4. — Interactive network analysis of DEPs between pregnant and non-pregnant ewes in the C area. Proteins enclosed in color-coded outlines are mainly involved in carbohydrate metabolism (red) and interferon signaling (green). [file 40104_2015_17_MOESM6_ESM.tiff]

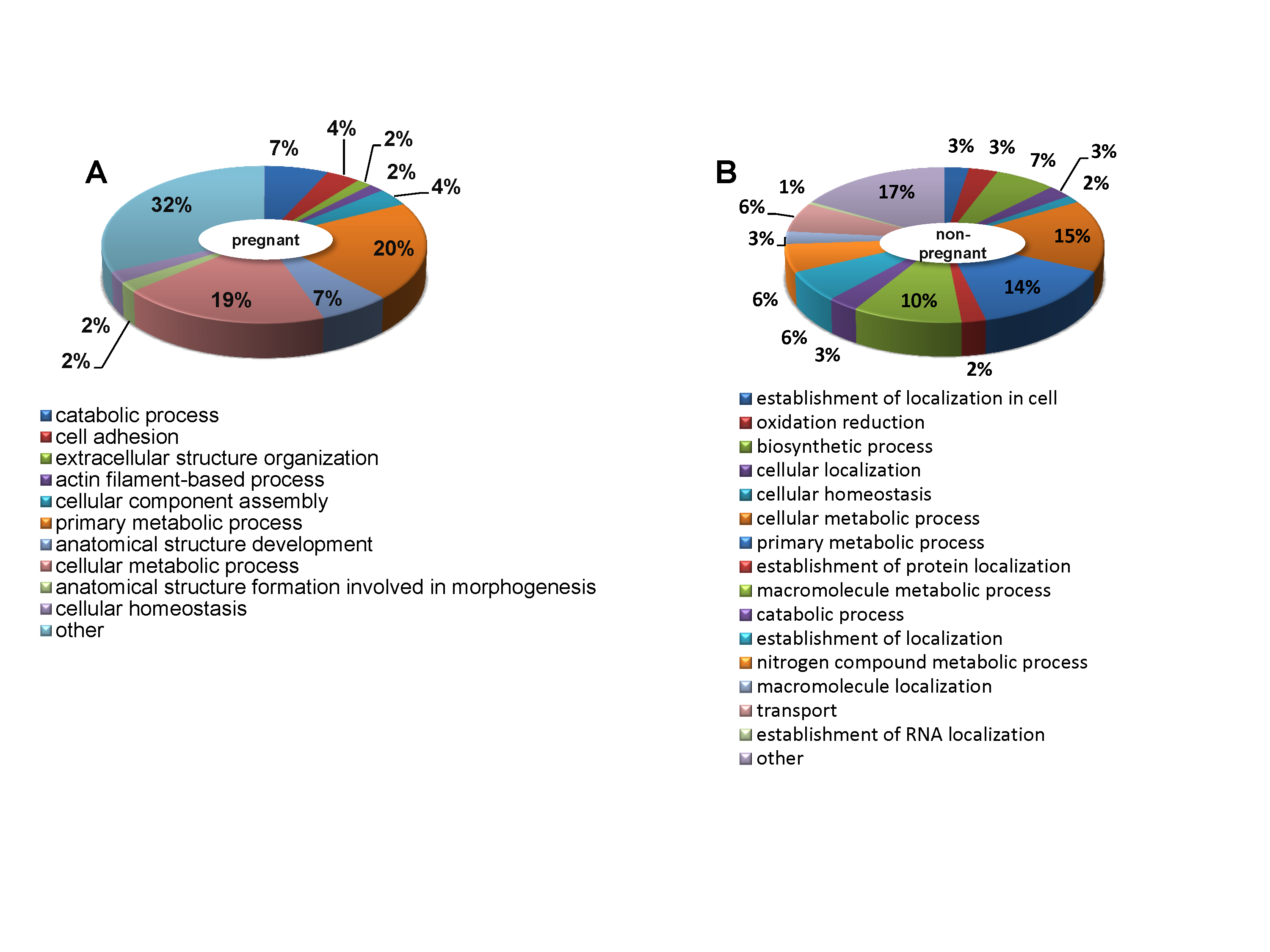

Supplement: Additional file 7: Figure S5. — Gene Ontology “biological process” classifications of DEPs (C vs. IC) in the pregnant (A) and non-pregnant (B) ewes. [file 40104_2015_17_MOESM7_ESM.tiff]

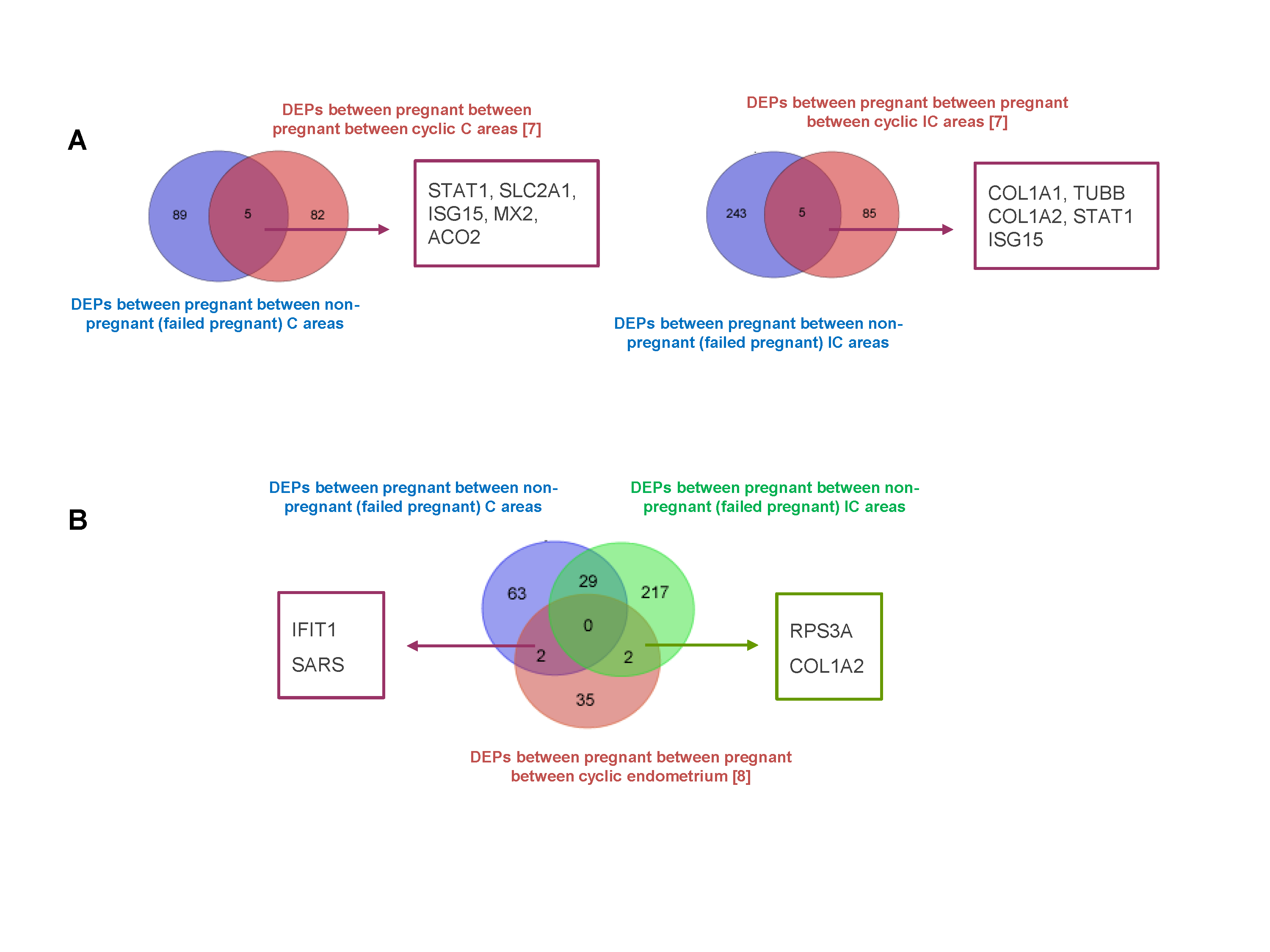

Supplement: Additional file 8: Figure S6. — Venn diagrams for retrospective analysis of DEPs in the present study with previously published related studies [7,8]. (A) Comparison of candidate proteins associated with failed pregnancy with gens responsible for pregnancy establishment in C and IC areas respectively [7]. (B) Comparison of candidate proteins associated with failed pregnancy in C and IC areas with endometrial genes responsible for pregnancy establishment [8]. [file 40104_2015_17_MOESM8_ESM.tiff]
